# A wise person plants a tree a day before the end of the world: coping with the emotional experience of climate change in Poland

**DOI:** 10.1007/s12144-022-03807-3

**Published:** 2022-10-14

**Authors:** D. Zaremba, M. Kulesza, A. M. Herman, M. Marczak, B. Kossowski, M. Budziszewska, J. M. Michałowski, C. A. Klöckner, A. Marchewka, M. Wierzba

**Affiliations:** 1 Laboratory of Brain Imaging, Nencki Institute of Experimental Biology, Polish Academy of Sciences, Warsaw, Poland; 2 Department of Psychology, Norwegian University of Science and Technology, NTNU, Trondheim, Norway; 3 Department of Psychology, University of Warsaw, Warsaw, Poland; 4 Laboratory of Affective Neuroscience in Poznan, Faculty of Psychology and Law, SWPS University of Social Sciences and Humanities, Poznań, Poland

**Keywords:** Climate change, Emotion, Coping strategies, Climate anxiety

## Abstract

It is now widely accepted that we are in a climate emergency, and the number of people who are concerned about this problem is growing. Yet, qualitative, in-depth studies to investigate the emotional response to climate change were conducted either in high-income, western countries, or in low-income countries particularly vulnerable to climate change. To our knowledge, there are no qualitative studies conducted in countries that share great barriers to decarbonization while being significant contributors to carbon emissions. Since climate change affects people globally, it is crucial to study this topic in a variety of socio-political contexts. In this work, we discuss views and reflections voiced by highly concerned residents of Poland, a Central European country that is a major contributor to Europe's carbon emissions. We conducted 40 semi-structured interviews with Polish residents, who self-identified as concerned about climate change. A variety of emotions related to climate change were identified and placed in the context of four major themes: dangers posed by climate change, the inevitability of its consequences, attributions of responsibility, and commonality of concern. Our findings highlight a variety of often ambivalent and conflicting emotions that change along with the participant’s thoughts, experiences and behaviours. Furthermore, we describe a wide repertoire of coping strategies, which promoted well-being and sustained long-term engagement in climate action. As such, our work contributes to research on a broad array of climate-related emotions.

**Supplementary Information:**

The online version contains supplementary material available at 10.1007/s12144-022-03807-3.

## Introduction

### Facets of climate change concern

Climate change is currently viewed as a major threat to the survival of species and the integrity of ecosystems worldwide (IPCC, 2021). Recent studies conducted in numerous countries—including USA (Leiserowitz et al., 2021; Marks et al., 2021), Australia (Marks et al., 2021; Morrison et al., 2018), United Kingdom, France (Marks et al., 2021; Steentjes et al., 2017), Norway, Germany (Steentjes et al., 2017), Finland, Portugal, Brasil, India, Philipinnes, and Nigeria (Marks et al., 2021)—suggest that people are aware of the dangers related to climate change and are increasingly concerned about this problem.

Because of its complex nature, climate change gives rise to a variety of emotions (Gardiner, 2011). Novel terms, such as *psychoterratic syndromes (“earth-related mental health issues”)*, *solastalgia (“the distress or desolation caused by the gradual removal of solace from the present state of one’s home environment”)* or *ecoanxiety (“anxiety related to a changing and uncertain environment”)* have been proposed to describe human reactions to climate change, environmental destruction, and related phenomena (Albrecht, 2011). The main focus of the psychological research on climate change concern is its potential impacts on mental health and wellbeing (Clayton, 2020; Cunsolo & Ellis, 2018; Ogunbode et al., 2021; Ojala et al., 2021). Strong emotional responses to climate change are often described with terminology sourced from clinical contexts, such as *climate change anxiety* (Clayton, 2020), *eco-anxiety* (Hogg et al., n.d.) or *eco-depression* (Stanley et al., 2021).

In opposition to that Marczak et al. (2022) explored what highly concerned people in Norway feel about climate change. Importantly, the scope of their study was not constrained to investigate how climate change impacts mental health, but also it examined the way climate change shaped various areas of participants’ lives. Their exploration revealed a complex panorama of emotions, which included: sorrow around the losses brought by climate change, anger at the perceived lack of engagement or deliberately harmful conduct of people in power, irritation and feelings of isolation around the perception that other people do not care about climate change, fear around the fate of future generations, despair about catastrophic visions of the future, guilt around feeling personal responsibility for climate change, as well as positive emotions, such as love for nature, or sense of community and empowerment around collective climate action. Thus, in this context strong emotions can also be regarded as appropriate, adaptive responses (Cunsolo et al., 2020; Verplanken & Roy, 2013), and shouldn't be treated as pathology (Budziszewska & Kałwak, 2022).

Indeed, a recent review by Pihkala (Pihkala, 2022) shows that the emotional experience of climate change is broad and diverse. Also, while many of these emotions seem to be appropriate and constructive responses, they can be challenging to cope with (Ojala et al., 2021).

### Coping with climate change concern

Coping is crucial for the psychological adjustment of an individual, but also from the societal perspective, because it can either foster mitigation and adaptation behaviours or result in the lack of involvement (Helm et al., 2018; Homburg et al., 2007; Homburg & Stolberg, 2006). Coping with climate concern has been studied mostly from the perspective of cognitive coping theory (Lazarus, 1991; Lazarus & Folkman, 1984). Researchers who proposed their tools to measure coping with global environmental problems (Homburg et al., 2007), agree that the following three main groups of coping strategies can be identified: emotion-focused, problem-focused, and meaning-focused strategies. *Emotion-focused strategies*, such as information avoidance, distancing, or denial, focus on the elimination of negative emotions evoked by climate change. *Problem-focused strategies* can take the form of information seeking or involvement in collective climate action to reduce the cause of the distress. In the context of climate change, the use of individual problem-focused strategies can lead to even greater distress because individuals have insufficient agency to eliminate the threat (Clayton, 2020). On the other hand, collective climate action has the potential to reduce distress and its consequences to mental health (Bradley et al., 2014), as well as to provide a sense of purpose in life (Bamberg et al., 2018; Corral-Verdugo et al., 2011). *Meaning-focused strategies*, such as cognitive reappraisal, can be used to regulate emotions by transforming negative feelings into positive feelings and, in turn, by changing one’s perception of climate change. Meaning-focused coping was found to be related to higher environmental engagement and efficacy beliefs, as well as higher levels of positive and lower levels of negative affect (Ojala, 2012a).

It has been found that coping strategies may differ in terms of their effectiveness and lead to different mental health outcomes (Gross & John, 2003; Nolen-Hoeksema & Morrow, 1993; Aldao et al., 2010). Moreover, flexibility in the use of coping strategies is central to well-being and resilience (Sheppes et al., 2014). Importantly, the choice of a particular strategy depends on the intensity of the stressful situation (Sheppes et al., 2011), individual predispositions and goals, as well as external circumstances, such as available resources that depend on socio-political, cultural or economic context (Clayton, 2020; Higginbotham et al., 2014).

### Socio-political context of emotional response to climate change

Most in-depth, qualitative studies on emotional responses to climate change were conducted in two contexts. Firstly, some research comes from low-income countries and areas inhabited by indigenous populations, that experience noticeable changes in their home environment and are especially vulnerable to climate change (Carleton, 2017; Cunsolo Willox et al., 2012, 2013; Furberg et al., 2011; Gibson et al., 2020; Middleton et al., 2020). Secondly, researchers targeted populations of high-income western countries, that are shielded geographically and economically from the imminent effects of climate crisis, but at the same time are open to addressing the need to mitigate climate change (Ellis & Albrecht, 2017; Head & Harada, 2017; Kemkes & Akerman, 2019; Marczak et al., 2022). Strikingly, there are no qualitative studies conducted in countries that are commonly recognized as significant contributors to worldwide carbon emissions, yet, due to many factors, share great barriers to decarbonization.

For instance, the gap in the literature exists when it comes to research on Central and Eastern European countries that used to belong to a group of socialist states called Eastern Bloc during the Cold War (Assetto, 2019). These countries are industrialised, but—due to the wealth gap between them and the high-income countries—they focus on economic growth at the expense of environmental destruction. The historically strong reliance on coal for energy production and the pressure of various interest groups pose strong barriers to decarbonization (Brauers & Oei, 2020). This can be illustrated by the fact that *per capita* greenhouse gas emissions in some Eastern Bloc countries (e.g. Czech Republic or Poland) are higher than the EU-28 average (European Environment Agency, 2021). Therefore, it seems crucial to study such societies, in which individuals concerned about climate change are aware of the resistance to decarbonization and transition to a greener economy expressed by their current governments.

Like many similar countries, Poland—a Central European country with a population of approximately 38 million—inherited its high energy- and carbon-intensive economy from the previous political regime (Li et al., 2020). Poland’s energy production is heavily reliant on harmful fossil fuels—lignite and hard coal, which produce nearly 70% of electricity (Statistics Poland, 2021). A power plant in Bełchatów, Poland was the world’s biggest single CO_2_ polluter in 2018 (Grant et al., 2021). Poland is the largest hard coal producer in the EU, and has not committed to ending coal mining anytime soon (Brauers & Oei, 2020). It has also vetoed EU’s policies aimed at increasing climate protection, arguing that the economic costs of such solutions would be too high (Brauers & Oei, 2020). In fact, Poland was the only EU country that opposed the goal of achieving climate neutrality in the EU by 2050 (Bohdanowicz, 2021). Despite this reluctance on a political level, a recent study demonstrated that support for climate policy in Polish society is just as high as in European countries with a more ambitious and progressive climate policy (Bohdanowicz, 2021).

### Current study

While most in-depth, qualitative research by necessity focuses on describing the perspective of a single, local community (Tschakert et al., 2019), the need to study other populations is widely acknowledged (Ojala et al., 2021). In the present study, we aim to extend the emerging body of knowledge on emotional responses to climate change studied in different socio-political contexts (Gibson et al., 2020; Kemkes & Akerman, 2019; Middleton et al., 2020). Building on earlier work conducted in Norway by Marczak and colleagues (Marczak et al., 2022), we contribute data from a novel context. We conduct our study in Poland, a Central European country, a large CO^2^ polluter, whose government resists the need for rapid decarbonisation. In particular, we explore major *themes* or *topics* evoking strong and potentially opposing emotions and highlight the active role of individuals in naming, transforming, regulating and coping with these emotions. Given the exploratory nature of the study, three overarching research questions were formulated:

1. What are the major topics associated with strong emotions experienced in relation to climate change in Poland?
2. What emotions are experienced specifically?
3. How do individuals regulate and cope with these emotions?

## Materials and methods

This study was a part of an international research project “Understanding patterns of emotional responses to climate change and their relation to mental health and climate action taking”. The first part of the research was conducted in Norway and the results are described in a separate publication (Marczak et al., 2022) . The excerpts shared in this article are drawn from 40 in-depth interviews with residents of Poland who self-identified as strongly concerned about climate change, collected between March and May 2021.

### Participants

Polish-speaking adults who expressed strong concern about climate change were invited to participate in the study. The recruitment was carried out through announcements in social media, mailings, and by word-of-mouth. 162 volunteers signed up for the study, out of which 29 had to be excluded due to incomplete data (e.g., missing contact information). Whereas sample size in qualitative research is primarily determined by data saturation, based on a previous, comparable study (Marczak et al., 2022), we expected that data saturation would be achieved with N = 40 participants. To ensure the diversity of perspectives, we selected participants based on purposive sampling. Participants (19 men, 21 women) were selected based on the order of registration, until a similar number of representatives for each gender and age group was achieved. The interviewees' age ranged from 18 to 82 years (M = 37.38, SD = 16.59), with participants representing various generations: Generation Z, Millennials, Generation X, Baby Boomers and Silent Generation (Table 1). Generation cut-offs were defined as by Pew Research Center (Dimock, 2019). Importantly, we intentionally selected participants with various demographic characteristics (e.g. place of residence, educational attainment, parenthood status). Moreover, we expected that people professionally related to the topic of climate change (e.g. scientists, climate activists) would express greater concern (Kleres & Wettergren, 2017; Wang et al., 2018) and we aimed to include such participants in the study sample. In the case of 10 participants who dropped out due to various reasons, additional participants were selected for the study. Participation in the study was voluntary and subjects provided informed consent. Participants were offered remuneration.

**Table 1:** The number of participants recruited for the study by age, gender, place of residence, education, parenthood status and environmental engagement

	Number of participants	
	Men	Women
*Age*		
Generation Z (M = 21.00, SD = 1.73, 18–24 y.o.)	6	5
Millennials (M = 32.71, SD = 4.82, 25–40 y.o.)	9	8
Generation X (M = 49.67, SD = 3.88, 42–53 y.o.)	2	4
Baby Boomer (M = 65.60, SD = 3.65, 61–70 y.o.)	2	3
Silent Generation (M = 82, SD n/a, range n/a)	-	1
*Place of residence*		
Urban (over 500 000 inhabitants)	22	
Urban (from 100 000 to 500 000 inhabitants)	6	
Urban (up to 100 000 inhabitants)	6	
Rural	6	
*Education*		
Primary	1	
Secondary	10	
Higher	29	
*Parenthood status*		
No children	23	
Children	17	
*Environmental engagement*		
Professionals (e.g. scientists, NGO, public services, or private sector)	13	
Climate activists	10	
None of the above	17	

### Data collection

The interviews followed a conversational format (Kvale, 1994; Kvale & Brinkmann, 2009). This approach allowed conversations to evolve spontaneously, avoiding constraints imposed by a fixed structure. The interviews were conducted based on an interview guide inspired by the materials used by Marczak et al. (2022). Importantly though, the interviewers could adjust the sequence of topics and add more questions based on the context of the participants’ responses. The interview guide can be found in Supplementary Table 1. Furthermore, interviewers used additional materials, i.e. Plutchik’s wheel of emotions (Plutchik, 2001). Interviewers explained that Plutchik’s emotion wheel presents emotion concepts that people usually use to describe various intensity and types of emotion. Participants did not have to elaborate on every emotion category, but could talk about emotions they found relatable. If needed, the interviewers offered assistance by rephrasing or clarifying emotion concepts.

The interviews were conducted by four team members (DZ, MK, AMH, MW), with each of them conducting around 10 interviews. The interviewers completed training to get familiar with the interview guide and attended weekly supervision with an experienced psychotherapist (JMM). This allowed the interviewers to reflect on how their personal experience of climate change might have influenced the interviews (Braun & Clarke, 2019). Participants were informed about the objectives of the study prior to the interview and the role and affiliations of the interviewers. The interviews were conducted remotely, either via online video conference or by phone, and audio-recorded with consent from the participants. Most interviews were conducted in one session and lasted from 28 to 152 min (M = 71.95, SD = 24.30). Importantly, the interviewing team met regularly to verify whether data saturation was reached and ensure that each researcher has built a thorough understanding of the topics raised by the participants. Indeed, after 40 interviews were conducted the interviewers agreed that data saturation has been reached and subsequent interviews would not contribute new relevant knowledge.

### Data analysis

Data obtained from the interviews was analysed in a rigorous, collaborative, and reflexive process (Braun & Clarke, 2006, 2019). Audio recordings were anonymized by removing fragments containing personal data, and by modifying the sound properties of the recordings. The recordings were transcribed *verbatim* by professional transcriptionists and the interviewing team (DZ, MK, AMH, MW). MAXQDA 2020 (VERBI Software, n.d.) was used for data annotation.

Several precautions were taken to ensure that the findings accurately reflect the collected data. Firstly, each of the four interviewers proposed their initial coding system, based on the content of two different interviews of their choice. The proposed initial versions of the coding system were compared and discussed between interviewers to ensure a common understanding of the final coding system. Secondly, each of the interviewers contributed to the development of the final coding system. If—in their view—some important topic was not covered, the interviewers could request a change in the coding system. Lastly, each interview was coded according to the final coding system by at least two interviewers. Importantly, each individual sentence or paragraph could be annotated with multiple codes. In cases of coding inconsistency, discrepancies were discussed and agreed upon within the interviewing team.

Once all the interviews had been coded, the data was analysed using a multi-step thematic approach (Braun & Clarke, 2006). First, an initial set of themes was identified, based on commonly co-occurring codes. Next, the initial themes were pooled together into more general themes. Importantly, themes were discussed by team members at all stages of the analysis to ensure that they accurately reflected the collected data. Finally, clear definitions and names for each theme were proposed.

### Positionality and ethics

The choice of the qualitative method was motivated by the aim to capture participants' views of climate change and the emotions stemming from it. As emotions are ambiguous and intangible, interviews are useful in this domain of research. In the present work, we have employed a reflexive approach (Finlay & Gough, 2003; Vestergren & Drury, 2020). The study was coordinated by a psychologist and a doctoral researcher (DZ), with a background in environmental psychology, neuroscience and clinical psychology. Data collection and analysis were conducted by two doctoral (DZ, MK) and two postdoctoral (MW, AMH) researchers. Each of the four interviewers belongs to the intersection of demographic groups systematically found more prone to experiencing concern related to climate change in western, democratic countries: climate scientists (Head & Harada, 2017; Wang et al., 2018), younger generations, and women (Lewis et al., 2019; Patrick et al., 2021). Furthermore, the results were discussed with team members experienced in environmental psychology (MM, MB, CAK), psychology of emotions (AM) and clinical psychology (JMM). All members of the research team describe themselves as concerned about climate change. To reduce personal bias we implemented certain steps during data collection and analysis (e.g. supervision of interviewees, group discussion of emerging themes). Thus, thanks to our contextual knowledge and personal understanding of the points of view present among individuals concerned about climate change we could come to a more informed interpretation of the collected data (Shaw, 2010).

The methodological details of our study are reported according to the consolidated criteria for reporting qualitative research (COREQ, Tong et al., 2007) and summarized in Supplementary Table 2. The study protocol was approved by the SWPS University of Social Sciences and Humanities Research Ethics Committee in Poland (approval no. 2021–52-12).

## Results

The thematic analysis revealed that interviewees experienced a variety of emotions towards different aspects of climate change. Many interviewees reported seemingly contradictory opinions and conflicting emotions as they touched upon different topics during the interview. Participants’ emotions changed throughout their life, along with their perception of climate change.

We identified four major themes with respect to which people felt strong emotions: the dangers of climate change, the inevitability of its consequences, attribution of responsibility, and commonality of concern. For each theme, we were able to identify two subthemes that represent opposite stances one could take in a given context. As demonstrated by our work, these opposite stances resulted in different emotions experienced by interviewees.

**Fig. 1:**
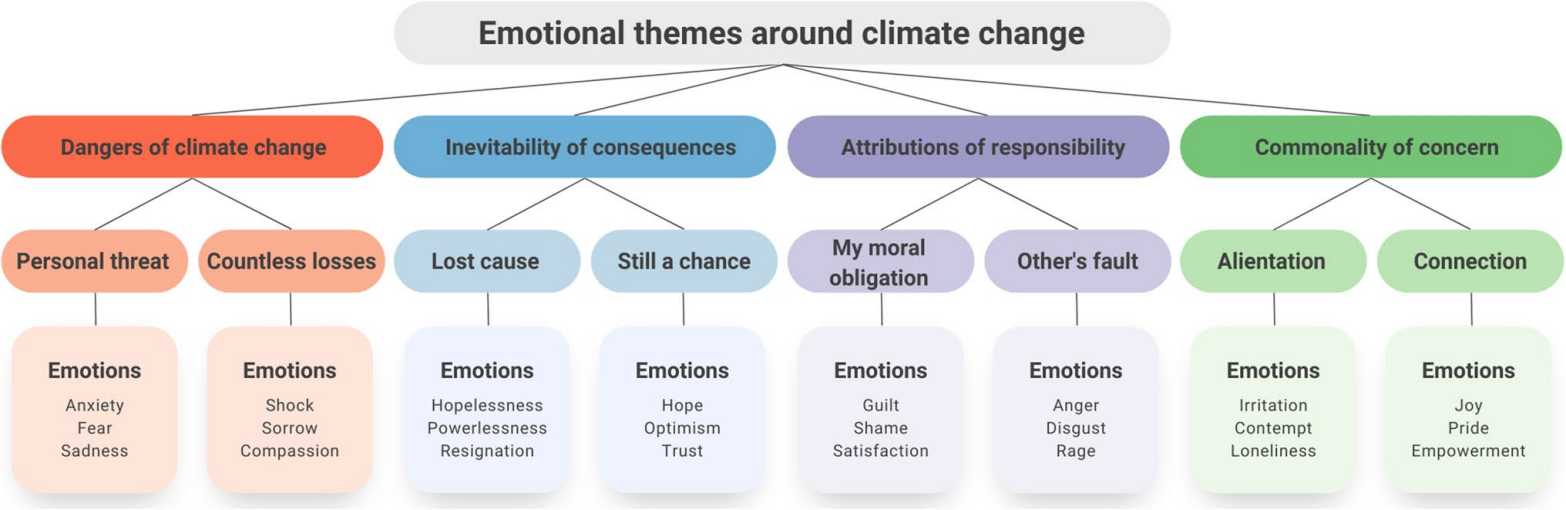
Emotional themes around climate change and the accompanying emotions

In the following sections, we describe each major theme, together with the corresponding subthemes. First, we describe the context in which emotions emerged (both external circumstances, as well as accompanying thoughts, experiences and behaviours), then we discuss the resulting emotions following how they were named, expressed and experienced by the interviewees, and finally we report coping strategies used to regulate these emotions. The following abbreviations are used to mark types of coping strategies: emotion-focused (EF), problem-focused (PF) and meaning-focused (MF). Figure 1 presents a general overview of the structure of themes and subthemes, and Table 2 provides more detailed information on that matter. Excerpts from the interviews used in this article are listed in supplementary materials.

**Table 2:** Emotional themes around climate change, along with examples of a context, emotions and coping strategies

Theme	Subtheme	Example of a context	Examples of emotions	Examples of coping strategies
Dangers of climate change	Personal threat	Observing environmental destruction happening nearby	Anxiety, fear, sadness	Information avoidance (EF), individual adaptation behaviours (PF)
	Countless losses	Watching nature documentaries about animals going extinct	Shock, sorrow, compassion	Contact with nature (EF), collective conservation efforts (PF)
Inevitability of consequences	Lost cause	Reading about the inefficacy of efforts to limit climate change	Hopelessness, powerlessness, resignation	Information avoidance (EF), Involvement in climate action (EF), Acceptance of ambivalent feelings (MF)
	Still a chance	Reading about a technical solution to the problem of climate change	Hope, optimism, trust	Information seeking in order to prevent being fooled by green-washing (EF)
Attributions of responsibility	My moral obligation	Engaging in climate-unfriendly behaviour	Guilt, shame, satisfaction	Individual mitigation behaviours (PF), reappraisal of responsibility (MF)
	Other’s fault	Reading about corporations harming the planet	Anger, disgust, rage	Collective climate action (PF), Excuse seeking (MF)
Commonality of concern	Alienation	Arguing about climate change with the family	Irritation, contempt, loneliness	Withdrawal from relationships (EF), engaging in organised activism (PF)
	Connection	Participation in the protest	Joy, pride, empowerment	–

The following abbreviations are used to mark types of coping strategies: *emotion-focused* (EF), *problem-focused* (PF) and *meaning-focused* (MF)

### Dangers of climate change

Climate change was perceived, first and foremost, as a major threat. On one hand, participants’ intense emotions resulted from the concern for their safety. On the other hand, they expressed strong concern for the survival of humanity, of other species and the integrity of ecosystems.

#### Personal threat

Participants experienced acute anxiety upon the realisation that climate change is a real danger to them and their families. Younger participants expected to experience climate crisis in the course of their own lives. Older participants did not expect to live long enough to experience the consequences of climate change, but they were extremely worried about the safety of their relatives. Climate change and its consequences—witnessed in person or seen in the news—were reported as disturbing and frightening. When asked about specific sources of fear, participants reported being afraid of extreme weather events, natural catastrophes, water and electricity shortages, food insecurity, forced migrations, wars and social disorder, mass extinction of species, and environmental degradation. For example, one participant said:

“I begin to notice abnormal weather patterns in Poland (…) hurricanes, sand storms (…). And the scale of this fury of nature fills me with a kind of dread.” (Woman, 51 y.o.).

Awareness of impending climate catastrophe undermined participants’ “basic sense of security” (Man, 37 y.o.). The feeling of apprehension was described as persistent and disturbing, always present “at the back of mind”:

“I think that this feeling will be following me all the time… no matter what. The thought that something bad will happen, something we cannot predict. This anxiety just never leaves me. Sometimes it is more intense, sometimes less, but it is always there.” (Man, 20 y.o.)

Uncertainty about the future in a highly unstable and dangerous world left participants paralyzed and unable to make plans for the future. Besides realising that their health and safety is threatened, participants felt responsible for making difficult decisions that could contribute to the suffering of other people:

“The moment when climate migrants will be standing at our borders and we will have to decide whether we are letting them in, and on what terms. I certainly fear such moral dilemmas.” (Man, 19 y.o.)

Participants anticipated their standard of living to get much worse. They felt upset when thinking about giving up on their plans that were incompatible with their concern for the climate. They talked about the need to let go of their plans for travels to places they always wanted to see, becoming a parent, or living a comfortable life. The topic of voluntary childlessness was also brought up in this context. Having a child in a dangerous or possibly uninhabitable world was considered unethical or irresponsible. One participant asked about how she imagines her future replied:

“I certainly see it without the children I might have given birth to. I could still adopt a child, but to bring children into a world that is coming to an end, it doesn't sit well with me.” (Woman, 21 y.o.)

Participants reported not being ready to face the reality of climate change, and they sought ways to adapt to its challenges (PF). They tried to reduce their exposure to disturbing information in the media to put their mind at rest (EF). Distancing from the threat was a way of protecting their mental health:

"I think we do need a lot of this kind of information… But, as I've said, to protect my mental health… I filter the news feeds quite heavily. I try… not to get too depressed by events around the world, because in spite of everything… On the one hand, I am aware that my knowledge of these topics is not complete, but I feel… that I know enough, that I don't need to make myself even more gloomy. I give myself permission to do this." (Woman, 22 y.o.)

#### Countless losses

Besides being worried about their own safety, participants felt sorry for other victims of climate change. In fact, participants did not expect to be personally affected by climate change to the extent that people in other parts of the world would. They viewed Poland as a relatively safe place to live and attributed their safety to its privileged location. At the same time, participants expected climate change would bring many losses for humanity, other species and the environment.

Participants mourned the degradation of the environment. Seeing beautiful sites being destroyed by people or polluted by industrial waste, left them feeling shocked and speechless:

“This entire sanctuary ecosystem began to die, right in front of my eyes (…) and it hit me really hard.” (Woman, 38 y.o.)

Climate change was seen as a phenomenon bringing death and destruction. Thinking about the victims of climate change—both humans and other species—made them profoundly sad. At the same time, they felt entrapped in a society that brought destruction upon itself and the planet. A number of participants couldn’t help crying and expressed their strong emotions during the interview when mentioning familiar natural environments—once beautiful and bursting with life, now being degraded due to human activity.

"So many species of animals and plants don't have habitats anymore. When I see the soil turning into dust, blown away by the wind I have a feeling that something is dying in front of my eyes. (...) In the name of what? (...) This is a bit like, I don't know, a drug addict, for example, who destroys their own body day after day in order to feel good for a little while longer. And we take part in this, basically whether we like it or not, which is even more sad." (Man, 53 y.o.)

Old, beautiful trees were mentioned on multiple occasions as prototypical victims of climate change and environmental degradation. Participants saw trees being knocked down by extreme weather events, but also damaged by humans. One of the participants described her compassion for the natural world as follows:

"I am aware of the pain felt by a tree, when I see healthy, beautiful trees that have been growing for a very long time, sometimes even several hundred years, cut down with a saw or an axe. It is as if someone were sticking pins in my heart. We mustn't do this! It hurts me very much. I feel sorry for the birds that are no longer there.” (Woman, 82 y.o.)

Participants acknowledged that these strong emotions made them realise how much they valued nature. They felt motivated to try to save as much as they could and engaged in individual or collective conservation efforts—for example collecting rubbish in the forest or donating to wildlife protection charities (PF). One participant, a forester, shared a story about a loss of a tree, that exemplifies the deep respect and love for nature shared by the interviewees:

"Some trees I cannot forget. (…) Here is what happened in [the name of a city] (…) There is now a shopping mall at the site, a huge one. And in the corner of the plot, two oak trees, one very old, a natural monument, the other younger, but very beautiful, a little bit further away. (…) The perfect successor to the old one. (…) The developer [responsible for the construction of the shopping mall] applied for a permit to remove all the trees. Some individuals and social organisations tried to stop it. But no. Can you imagine? The oak was finally cut down (…). I have to say, I still do not understand… I do not understand the ruthless perseverance with which people wanted to kill this tree. (…) I cannot forgive this. I have never been there and I will never visit this mall. I just… I am so angry about this… (…) I suppose I have this deep conviction that… I suppose I see trees as… as individuals, as persons. That's why it moves me so much." (Man, 61 y.o.)

In times of overwhelm, participants also sought refuge in being able to enjoy nature in the present moment (EF). They found contact with the natural world soothing and restorative, as described by one of the participants in the following words:

"Euphoria, joy, serenity… it was definitely during the pandemic that we became aware… that I became aware of how much... how much more than before, I enjoy going out for a walk… in my city, for example… along the river, in the bosom of nature… It gives me serenity, joy and… momentary freedom from negative impulses that go with climate change… Just to enjoy nature, to experience it…" (Man, 20 y.o)

### Inevitability of the consequences

Climate change was believed to have a decisive impact on the future of the planet and human civilization. On one hand, participants grew hopeless the more they learned about the failed efforts to stop climate change. On the other hand, they kept finding reasons to sustain hope, despite realising that many consequences of climate change are inevitable.

#### Lost cause

The continuous rise of global greenhouse gas emissions despite individual or collective efforts made participants think that humanity is unable to stop climate change. The societal ignorance towards the environmental issues and lack of urgent and decisive action on the systemic level made them feel pessimistic about the future. One participant shared her perspective using the following words:

"I look at the world with hope that maybe we can still stop the climate catastrophe, but if nothing changes, I am not optimistic. So, when I think about this, the first time this struck me with such force, was when I saw (…) a man with a banner that read “It terrifies me to think I will be capable of killing for water when climate war breaks out.” This made me think that indeed if this happens, I do not wish to live in such a world. It would be better to jump from a cliff than to be part of it." (Woman, 21 y.o.)

Participants felt disillusioned, resigned, powerless and hopeless when asked about the future. In their view, irreversible environmental destruction and societal collapse were unavoidable. Furthermore, they often mentioned various physical symptoms such as sensations of numbness, light-headedness, weight on the chest and in the limbs, as well as lower energy levels:

“I was definitely feeling down. I really felt there was nothing for me to do but sit and cry. Actually, I'm aware of the situation and that really bad things will happen. I do not fool myself that things will change because I think they will not change. We really are about to run into a brick wall headfirst. (…) In fact, there are not many situations that make me so drained of energy, that I don’t have strength to act, it is very rare for me.” (Woman, 42 y.o.)

Despite the high intensity of these emotions, most participants did not feel impaired in their daily functioning for prolonged periods of time, and just one out of forty participants sought psychological intervention because of their concern for climate. Those participants who received psychological treatment for other reasons admitted that their hopelessness related to the climate crisis contributed to their lower wellbeing. However, most interviewees reported that confusion, powerlessness and hopelessness were overwhelming only temporarily. Intense and paralysing emotions lasted from several hours to several days, but the milder forms of resignation or cynicism tended to return and stay for weeks.

Participants tried to cope with their strong emotions in various ways. Many interviewees spoke about how they tried to adapt to the reality of climate change. Climate change profoundly influenced their life choices: some took rising sea levels into consideration when buying building plots (PF), and one participant reported having picked the topic of studies in order to learn how to effectively mitigate climate change (PF). Still, they were acutely aware that their individual choices and decisions were not enough. While participants tried to distance themselves and avoid disturbing news, this brought only partial relief (EF). The feeling of hopelessness came back and forced participants to reinterpret climate change and their personal relation to it. For instance, participants spoke about climate change as just a punishment for humanity or as a natural or divinely planned course of events (MF). One participant described, how their spiritual perspective allowed him to distance himself from his experience of climate change (MF):

"It is a matter of trust and faith. In the spiritual sense, I certainly… trust the unknown and the benevolent powers that are present in the world and that can be experienced. This in turn is relevant to environmental thinking. For some, a sense of identity with the ecosystem is equivalent to religious faith. In any case, something that helps one distance oneself from individual existence or non-existence, from individual pleasure, pain, hope and fear. As if to adopt a larger perspective, because that's what is really needed. This is the challenge of these environmental times – to stay in touch with a wider perspective." (Man, 53 y.o.)

Despite being aware of the inefficacy of their efforts to stop climate change, to feel better some participants decided to participate in climate action despite all odds (EF). They spoke about how this decision gave them a source of meaning and empowerment:

“If not now, then never. And if we do not do it, then no one will. I know that activists are somewhat susceptible to illusions of omnipotence. But inaction... I despise inaction. That no matter what, even if it [confrontation with police during climate protests] is unpleasant and even if I am scared, I still have to do it, because otherwise I will not be able to achieve peace of mind and I will lose all self-respect. This also helps me overcome negative emotions." (Woman 23 y.o.)

The paradoxical, sustained involvement in the face of lost hope stemmed from participants’ personal values and moral choices (MF). One of the participants said he would continue his involvement in climate action even though the chances for “saving the planet” were very low:

"I am increasingly aware that, most likely, this planet will not be saved. I mean, it sounds pessimistic, but in fact this vision does not take away my motivation or make me feel depressed or something, I mean, it does not deprive me of the will to act, on the contrary. A wise person plants a tree a day before the end of the world. It is a kind of paradox, but that is how I try to live now.“ (Man, 51 y.o.)

#### Still a chance

In contrast to the feeling of hopelessness that was always “in the back of one’s mind” (Man, 26 y.o.), hope was like a dim “light in the tunnel” (Woman, 51 y.o.) that flashed occasionally. It appeared when participants heard about promising political and technical solutions being implemented. However, hope grew strongest when participants spoke about protests and demonstrations, such as Youth Strike for Climate.^1^ They admired activists’ dedication, courage and resilience. Their involvement in protecting the environment was seen as authentic and inspiring. In contrast, climate summits were considered as empty political shows and thinking about them was disheartening.

Participants shared a conflicted attitude towards their own hope. On the one hand, they needed hope to protect their mental health and stay engaged in pro-environmental behaviour. On the other hand, they disapproved of naive hope that technological advances would protect humanity from the problem of climate change, without significant sacrifices. Aware of the multiple forms of greenwashing (Lyon & Montgomery, 2015), participants remained sceptical about the good news.

“Hope? You know, if we manage to do anything about our ridiculously moderate targets, if we achieve these goals, our chances are still 50-50. So, in this case, it is hard to speak of any great optimism. We know that life will be difficult and, as far as optimism is concerned, I am just glad that there are people in the world who are more and more aware, and that there are countries, such as New Zealand, which enact new laws and pay attention to the environment, and that Joe Biden became president is also something positive. This is something really positive for the world, otherwise we would have totally hit the rock bottom. These are the only circumstances that inspire some optimism, but I know that it is still not enough, considering what is happening in the world at this moment.” (Woman, 30 y.o.)

Even though participants desperately looked for reasons to stay engaged, they did not want to experience ungrounded hope, dissonant with their goals and values. They used various strategies to cope with these unwelcome feelings. To sustain an informed, rational, constructive hope and avoid being fooled, they dedicated extra time and effort to cross-check every piece of information that could come out as greenwashing (EF).

Furthermore, they harshly judged themselves for feeling what they considered excessive pride or self-satisfaction with their climate-friendly behaviour (EF). Some participants lost hope that climate change could ever be stopped, but they still expressed trust in nature's and humanity’s ability to adapt to the new circumstances. Others doubted that the ambitious emission reduction goals could be achieved, but to deal with the risk of disappointment, they hoped for partial success:

"I believe that things will surely be bad (...), but they could be worse (...). If we continue to emit the same amount of carbon dioxide as we are emitting now, then, by 2100, temperature on Earth may increase by 4.5 degrees, and it is known that this would kill the Earth. If we apply the brakes and these targets are achieved at least in part, then maybe temperature will rise by 2.5 degrees, so there will still be something to fight for and every fraction of the degree on this thermometer counts." (Woman, 42 y.o.)

Participants viewed the upcoming decade as the last chance to effectively act on limiting the consequences of climate change. Tying up the hope to this tangible timespan, one participant said, that "There will be time to cry when it is too late to act." (Man, 21 y.o.).

### Attributions of responsibility

Responsibility for climate change was another theme which evoked strong emotions. Participants realised that they were contributing to the problem and felt morally obliged to act. At the same time, they placed the heaviest burden of responsibility on the privileged and powerful, on politicians, corporations, and the media.

#### My moral obligation

Since participants' attitudes towards climate change stemmed from their core ethical values, behaving in a climate-friendly way was seen as a moral imperative. One participant, reflecting on his own attitude towards climate change, said:

“[Climate change] for me has two sides. Positive and negative, because... By positive I mean... I can reflect on my morality... In fact, it is a question of morality for me, how we act about climate. This is my, I don't know, decalogue, if you will. It suits my spirituality, I guess... And I don't think that's too strong a word.” (Man, 20 y.o.)

Both day-to-day choices (such as shopping, commuting or recycling) and significant life decisions that could have a negative impact on the environment resulted in feelings of pride and satisfaction, or shame and guilt. Out of their concern for climate, interviewees engaged in (or forwent) various activities. However, this brought them only temporary relief (PF). These comforting feelings decreased over time, as participants solidified their climate-friendly choices into habits or found out how negligible their real impact was. Other participants denied experiencing satisfaction or pride because they regarded that as an unjustified complacency. In fact, the desire to avoid guilt, rather than the sense of satisfaction motivated their sustainable behaviour:

“As to my vegetarianism (…) even if I know that the [blame] is not all on me, it was such a small effort to make that it would have been embarrassing if I had not done it, considering how many meat substitutes are now available and how easy it is to quit consumption. That's why I decided to take this step, and it brings me a kind of relief.” (Man, 21 y.o.)

Despite efforts, participants often struggled between resignation and overwhelming guilt. One interviewee admitted to feeling ashamed for “living comfortably and for contributing with [her] lifestyle to the suffering of millions of people around the world” (Woman, 23 y.o.). Participants were far from perceiving their personal actions as sufficient to stop climate change, yet they were doubtful about being able to change their lives even more. In some cases, this feeling of guilt made the participants struggle with low self-esteem or even self-hatred:

"I do what I can, and I believe I try not to go to extremes, but yes, I do live in constant remorse. Sometimes I reach such levels of absurdity because of this remorse that I go back to the blue garbage can, the one for waste paper, and I take out the staples because I realised that I threw out the newspaper with staples.” (Woman, 38 y.o.)

One of the strategies used to relieve the guilt stemming from unsustainable behaviour was the denial of responsibility (MF). Participants were aware of inequalities in cumulative carbon emissions between countries and found their burden of responsibility for mitigation unfair. Poland was pictured as a country, whose citizens only recently began to experience a standard of living comparable to countries representing the western world (e.g. international travel, availability of material goods). Interviewees felt that they had the right to simply enjoy their lives, even if at the expense of the environment:

"I feel an emotion I'd call “catching up”. Doing things I haven't done yet… In the sense that there are places I'd like to see one day, because otherwise I wouldn't feel fulfilled, not entirely. So, I wanted to see various [places] and I still want to… I'm not saying that when I get vaccinated, I will fly around the world like a madman, but there are a few destinations I would like to visit. I read somewhere that it is hypocrisy to be a committed activist, and at the same time to fly on vacation. Still, I believe that if one has not done it up to a certain point in one's life, then it is permissible, because, for example, people in the West who pledge not to fly for the rest of their lives, have been doing it for 20 or 30 years, right? But we haven't. I mean, I don't know about you, but I haven't. I mean, much less than a statistical German, Englishman or Frenchman." (Man, 34 y.o.)

Another way to cope with unwelcome guilt was to accept one’s limited agency (MF). Participants pointed to the popular (but false in their view) narrative of individual responsibility for climate change, that they received from school, media, government and corporations. Yet, they were aware that these institutions had an interest in sustaining the *status quo*. One of the participants asked whether she feels guilty about climate change responded zealously:

“It is not alright to blame only individuals for what's happening, without taking into consideration the political context of where these people live, in what conditions, in what region of the world and so on. It's not alright that everything is being inverted, and that corporations and neoliberalism basically exploit such environmental and climate change discourse in order to instil a sense of guilt. I don't accept it. I just don’t. That's what I don't agree with because I think that's distorting the facts, reversing the truth.” (Woman, 32 y.o.)

The realisation that accepting the blame was in the best interest of the true actors responsible for climate change allowed participants to rebel against the voice of guilt and demand systemic change.

#### Other’s fault

Participants spoke about actors responsible for climate change: people in power, politicians, corporations, and the media. They claimed these actors propped each other's credibility. For instance, in their view, corporations could harm climate legally thanks to corrupt politicians and media spreading fake news about climate change. The intense feelings—moral disgust, rage and hostility—were directed toward the most powerful and influential politicians. One participant described their reaction as almost visceral, saying: “When I listen to [the president of Poland] I basically want to puke and I am full of contempt.” (Woman, 42 y.o.).

Besides that, some participants were disappointed with the Catholic Church and its influence on policy-making in Poland. Catholic Church was supposed to provide moral guidance in dealing with the problem of climate change, but instead, it was viewed as staying indifferent or even contributing to its disastrous effects (e.g. supporting the government's pro-coal policies). Even if participants noticed important religious leaders taking their side, they viewed such cases as incidental. One participant remarked that the teachings of Pope Francis stressing the need to protect the environment received very little publicity in Poland.

Regardless of who they blamed the most for climate change, participants pointed out the injustice resulting from it:

“Quite often I feel angry. I am convinced that this [climate] crisis has its fathers and mothers, rather more fathers. And these are flesh-and-blood people, with names, with wealth accumulated from certain activities, which, on one hand, has had a negative impact on the current situation. On the other hand, [this negative influence] has been covered up for many decades, as demonstrated time and time again by researchers. So, I am pissed off that, most likely, these people will avoid responsibility, maybe not even live to see the effects of their actions.” (Man, 35 y.o.)

Furthermore, participants blamed ordinary people for climate change: their relatives, colleagues, neighbours, or society in general. They were annoyed and outraged by people’s attitudes toward the environment and their careless, unsustainable behaviours. One participant racily described her irritation with environmental pollution:

“For example, as I once drove up to work, I saw the waste bins scattered about, I mean such utter thoughtlessness, which harms the environment on the local level, it really pisses me off. This is my hot button. When I see that someone – even though I know perfectly well that this individual behaviour, when extracted from the totality of human behaviour, has no effect on anything whatsoever – that someone acts without thinking and fails to take care of the local environment, it annoys me terribly, sets me off.” (Woman, 38 y.o.)

For some participants, this fundamental disagreement with others was hard to express and deal with. To protect themselves, they usually avoided direct confrontation with others (EF). Sometimes they made excuses for others' behaviour, quoting their poverty or lack of education (MF). Contrary to that, some participants transformed their strong emotions into climate action, as in their view this was “a kind of circumstance, in which the anger shouldn't be suppressed” (Woman, 23 y.o.). They enjoyed the energy that these strong emotions installed within them and they were eager to use these emotions in a productive way (PF):

“From time to time, I enjoy getting really mad and let something piss me off to the limits because I feel energised. And, to be honest, it is my favourite state of mind, a state of anger. And, well, when I hear something said by some politician or some, well, someone who denies all the bad things that are happening on Earth, or someone who is just questioning... Well, then I get mad, or even in my church, I get mad… And then I write something. Or take part in some action. So, I really like this state of anger.” (Woman, 68 y.o.)

### Commonality of concern

Finally, climate change was perceived as a highly divisive topic. Interviewees often felt alone and isolated in their strong concern for climate and paid the emotional cost of sustaining relationships with people whom they fundamentally disagreed with. At other times, they reported a sense of community with people of a similar mindset, and this motivated them to keep going and stay engaged in climate action.

#### Alienation

Many participants felt obliged to educate other people about climate change. However, they also noticed that this had a considerable impact on their relationships. They spoke about investing a lot of energy and emotional labour, trying to persuade others to change their behaviour and views on climate change. When they did not see the same effort coming from the other side, they felt lonely and misunderstood. Sometimes participants’ serious approach to the climate crisis gave them a label of a killjoy or crank. One participant described being torn between maintaining her relationships and acting in line with her personal values:

“This is again such a dichotomy, because, on the one hand, I would like to maintain important relationships, though I do not approve of the choices these people make every day. On the other hand, I do not want to be seen as an idiot, a loony, a mad person, or a crazy girl who, I don't know, has completely lost her sense of reality. I care about the impression I make. I do not want to be seen as someone crazy. And maybe I am. For who else would wage a campaign against plastic boxes?” (Woman 38 y.o.)

To prevent being perceived as fanatics, participants avoided voicing their opinions. Even when the interlocutor’s words infuriated them, they tried to hide their emotions and tame the impulse to correct the wrong view (EF). Many participants were tired of the open conflicts or keeping composure, which resulted in distancing or completely withdrawing from relationships (EF). One of the participants spoke about her inability to reach a deep, authentic understanding with her family because of her personal engagement in the topic of climate change:

“Well, I am trying to diagnose it myself, whether I am contributing to it [the distance]. It's as if I no longer care about my relatives so much. It used to be otherwise. I needed them very much every day. And now it makes me so sad because they know that I distribute my petitions, and they sign them, that is true. They read what I write, maybe not meticulously, but they know what I do. And as I've been saying, it's not that I've been cut off by anyone or that someone said to me “you are a complete freak, calm down”. However, it is painful that they do not share the same sensitivity, people I know, good people, sensitive people. And I have drifted away, and I look at them from afar, as if they belonged to a different human species.” (Woman, 68 y.o.)

The lack of support from the people they most cared about (e.g. family, friends) was even more painful than the awareness of climate change itself:

“I have reached a certain level of reflection myself, and other people, in many cases people close to me, do not support me. This disapproval… also leads to sorrow and grief. And the despair we talked about earlier. I can see it now. Yes. Not climate change by itself, but the disapproval… Realising that other people often think in ways quite opposite to mine. And that people that are close to me do not support me in my actions.” (Man, 20 y.o.)

Their social circle became narrower, as they severed relations with people whose views they could not accept. As a result, they felt they “lived in a bubble”, associating only with like-minded individuals, avoiding controversy and irritation. Bubbles offered a safe space to talk about one’s concerns without the risk of rejection. People with similar concerns were most frequently found on the internet or in climate activist groups, such as Youth Strike for Climate or Extinction Rebellion.^2^ Getting involved in collective climate action was an opportunity for self-development and life in harmony with one's values, as well as a source of support and a sense of acceptance within a community (PF).

#### Connection

Many intense, positive emotions resulted from interacting with other people concerned about climate. For instance, interviewees shared their experiences of joining climate strikes:

“I took part in the climate strike in England and it was probably the best thing that ever happened to me, because the people around me felt such strong emotions, quite indescribable. No concert or anything like it can reach the level of emotions that I experienced during this strike.” (Woman, 30 y.o.)

Similarly, joining an activist group was a source of empowerment. Participants spoke about the enthusiasm and energy of their fellow activists:

“The most emotional aspect of [name of organisation] is what we might call the romantic rebellion, something we learn about in a dull way at school, but here we see it actually happening. So, this is what generates a lot of strong emotions. I believe these are simple, pure emotions, joy, and satisfaction derived from the action. Experienced together, I mean, collective.” (Man, 21 y.o.)

According to the participants, interacting with other people motivated them to stay engaged despite the doubts they had about the efficacy of their own efforts. Even everyday chores, like shopping at a farmers' market from trusted and environmentally conscious sellers made the participants excited and happy.

Participants spoke about how becoming a member of a community or having a soulmate fulfilled their need for belonging, trust, and being accepted and understood. One participant spoke warmly about one of her friends:

“She also goes to various protests. It is so supportive, also psychologically supportive, because I don’t feel so alienated, so weird, like someone whose concerns are ignored by others as unimportant.” (Woman, 42 y.o.)

This need for belonging was—apart from the concern for climate—one of the strongest motivations for collective climate action. It was a source of empowerment and positive emotions, like joy, awe and pride. Sharing climate concern with other people improved participants’ well-being and helped them face the reality of climate change. Some activist groups offered support for people dealing with difficult emotions, fostering the culture of regeneration.

Even if the interviewees were not members of any organisation themselves, they were happy to see other people joining them. When participants felt isolated in their concern, thinking about activists who work to mitigate climate change helped them restore a sense of community that shares common values. One participant pointed out: “In fact, in the loneliness that I experience… I am not really alone.” (Man, 20 y.o.). Another participant said:

“I think it helps me and maybe it would help others too, just to pay attention to such organisations, which are active, because it gives you hope that you are not the only one who cares about it, that other people worry, too, and not just sit and worry, but actually do something to change it.” (Woman, 27 y.o.)

## Discussion

While in-depth, qualitative research conducted so far provided valuable insights, little is known about the experience of climate change in countries that resist decarbonisation, even though they are significant contributors to worldwide carbon emissions. Therefore, in this work, we have presented the voices of highly concerned residents of Poland.

### Emotional complexity and ambivalence

In the present study, we were able to document a wide spectrum of emotions experienced in relation to four major themes: dangers posed by climate change, the inevitability of its consequences, attributions of responsibility, and commonality of concern for the climate. The emotions reported by our participants were similar to those found in the previous studies (Marczak et al., 2022; Pihkala, 2022). In fact, recent research emphasised the need to formulate a taxonomy of climate emotions and the importance of taking these various emotions into consideration when discussing how climate change is experienced (Pihkala, 2022).

Anxiety, fear and sadness related to the “Personal threat” subtheme can be described as stemming from the egoistic environmental concern for one’s own and one’s family’s health and safety (Helm et al., 2018; Wesley Schultz, 2001). Although Polish participants could use these emotions to get energised towards climate action, unmanaged anxiety had a detrimental effect on their mental wellbeing. In fact, anxiety resulting from perceiving climate change as a “Personal threat” and hopelessness in the face of “Lost cause” are traditional targets of *climate anxiety* research (Clayton & Karazsia, 2020).

Sorrow and compassion described in the “Countless losses'' subtheme are experiences similar to those referred to as *ecological grief—*suffering related to losses of valued species, ecosystems and landscapes (Cunsolo & Ellis, 2018) or *solastalgia*—“the distress or desolation caused by the gradual removal of solace from the present state of one’s home environment” (Albrecht, 2019, pp. 38–39). These emotions seem to result from altruistic and biospheric environmental concerns (Helm et al., 2018; Wesley Schultz, 2001). The way Polish participants spoke about witnessing environmental losses resembled the accounts of indigenous people in Arctic Canada (Cunsolo Willox et al., 2012) or farmers in the Australian Wheatbelt (Ellis & Albrecht, 2017). Interestingly, many participants told stories about trees as examples of such environmental losses. The protection of trees became a subject of debate in Poland after the government introduced a bill allowing people to fell trees growing on private property (Kronenberg et al., 2021). Public protests eventually led to the repeal of the bill, but the situation encouraged many people to pay more attention to the presence and the condition of trees around them.

The “Lost cause” subtheme was connected to hopelessness and powerlessness, which were often accompanied by various physical symptoms felt in the body. Climate change was perceived as the ultimate existential threat, limiting the ways in which individuals can find a sense of purpose in spite of their mortality (Lifton & Jay, 2017). Because of climate crisis people are facing the death of the natural environment (for generations a permanent reality, continuing to exist despite the passing of individual human beings) and a real possibility of loss of their “symbolic immortality” (in a form of offspring and creative efforts, that may not survive on an uninhabitable planet) (Pihkala, 2018).

In this case, caring for the environment becomes a way to preserve one's own symbolic immortality and a source of hope in spite of the tragedy (Pihkala, 2018). Similarly to Swedish high school students in Ojala’s studies (Ojala, 2012b, 2015), our participants exhibited a complex relationship with hope. The “Still a chance” subtheme demonstrates the reserve in their optimism and elaborates on two types of hope. The first type, which participants wanted to avoid, was based on denial and caused complacency, while the other, constructive hope focused on trust in science, solutions and promoting involvement (Ojala, 2015).

Climate change—due to its anthropogenic nature—has been called a “perfect moral storm” and an ethical challenge for humanity (Gardiner, 2011). Indeed, participants reported experiencing many moral emotions. In particular, they spoke about guilt, shame, and self-satisfaction when they reflected on climate action as their own “Moral obligation”, or about rage, anger, and moral disgust when they considered climate change as “Other’s fault”. Participants differentiated two types of moral responsibility: the responsibility for causing climate change and the responsibility to engage in mitigation efforts. Participants placed the responsibility for causing climate change mainly on the people in power, institutions and the system as a whole. Such anger towards “destructive compulsions of techno-industrial civilization” has been described before with the term *terrafurie* (Albrecht, 2019). Responsibility for mitigating climate change was, however, placed on everyone: institutions, politicians, society, and the participants themselves. Similarly to the interviewees in Norgaard’s study in Norway (Norgaard, 2006a, 2006b), Polish participants sometimes resorted to denial to avoid their own voice of conscience when they behaved unsustainably. A particular, unique form of denial, related to the historical socio-economic situation of Poland was observed. Sometimes participants felt “entitled to emissions” because Poland as a part of the Eastern Bloc was for a long time cut off from the western standard of living. This type of interpretative denial might be prevalent among similar countries in the region that recently experienced rapid economic growth.

In the “Alienation” and “Connection” subthemes we described two facets of climate change as a topic of social debate, that can either divide or unite people. Participants reported feeling irritation and contempt towards people indifferent to climate change. Similarly to climate scientists in a previous study by Head and Harada (Head & Harada, 2017), Polish participants engaged in emotional labour to change other people’s attitudes towards climate change. The way one’s concern for climate influenced one’s relationships and the ability to fulfil the need for belonging was described previously by Marczak (Marczak et al., 2022). However, while participants in the current study felt similarly isolated in their views, they expressed more understanding for other people, who due to lack of environmental education and economic hardship wouldn’t prioritise climate change. They also found themselves contributing to those relational barriers: by trying to impose their views on others or breaking up the relationships. Luckily, many participants found a sense of belonging by engaging in climate activism, similarly to the participants in Pickard’s study (Pickard et al., 2020). They reported a variety of positive emotions related to climate activism, such as joy, excitement, pride, awe.

Participants struggled between conflicting or opposite emotions towards different aspects of climate change. In the course of the interview, participants often spoke from the perspective of contradictory positions described by different subthemes. Janet L. Lewis described similar internal conflicts in psychotherapy patients who experienced “an inability to properly contain (…) complexity and uncertainty” of climate change while wanting to formulate an individual response to a “problem unsolvable at a personal level” (Lewis et al., 2020). In other words, participants could not name a dominant attitude or a feeling toward climate change, because of the complex ways in which it challenged their well-being, decision-making, relationships and sense of responsibility. Touching on different topics prompted participants to describe their internally conflicted emotions and attitudes. They used various coping strategies when they experienced emotions related to a particular side of the dilemma, but also to transcend the conflict and reach a new, synthetic position.

### Coping strategies

We found that a wide variety of emotion-, problem-, and meaning-focused coping strategies (Lazarus, 1991; Lazarus & Folkman, 1984) were used by our participants to regulate their emotions related to climate change. Interestingly, participants often sought to use strategies that allowed them to improve their psychological wellbeing and sustain pro-climate engagement at the same time. Similarly, earlier studies found that problem-focused and meaning-focused coping strategies were positively related to pro-environmental behaviour (Homburg & Stolberg, 2006; Ojala & Bengtsson, 2019).

Our participants frequently used various problem-focused strategies. Individual climate mitigation behaviours helped to reduce guilt and the feeling of unpreparedness. Collective climate action improved participants’ well-being, was a source of empowerment and many positive emotions, like joy, awe and pride. Furthermore, collective climate action was seen as a possibility to experience a sense of meaning and belonging.

Meaning-focused coping strategies, such as self-distancing (Wang et al., 2019), cognitive reappraisal, mindful acceptance of negative emotions and appreciation for the positive experiences (Folkman & Moskowitz, 2007), allowed participants to distance themselves and change their perception of climate change. By looking at climate change through the lens of spirituality, they could accept their own mortality and the fact that human civilization may eventually end (Pihkala, 2018). Because of that, participants were able to simultaneously feel hope and hopelessness.

Emotion-focused strategies helped at the time of the greatest overwhelm and allowed the participants to regenerate. Interestingly, not only negative emotions were subject to regulation. Some positive emotions, like false hope (e.g. hope based on denial, Ojala, 2012b) and warm glow (Hartmann et al., 2017) were in conflict, or dissonant with the goals and values of participants. In order to prevent themselves from experiencing false, ungrounded hope, participants educated themselves on the practices of corporate greenwashing (Lyon & Montgomery, 2015) and sceptically approached novel, technological solutions to climate change. In fact, our participants highlighted that their sustainable behaviours stemmed from their deeply-held values and harshly judged themselves for feeling self-satisfaction or pride.

Finally, some participants did not want to escape from their negative emotions related to climate change. Sadness, anxiety and anger were perceived as congruent with participants’ values and provided motivation to act. In other words, participants recognized their personal engagement with the issue of climate change as a source of both pain and meaning.

## Conclusions

In this exploratory study, we have mapped out the emotional experience of climate change among Polish residents concerned about climate change and shown that the complexity and ambivalence of the emotions stems from the very nature of climate change—threatening, uncertain, unjust and socially dividing issue. Most of the time, participants successfully coped with their strong emotions in ways that also energised them to stay engaged in sustainable behaviour and climate action. Participants portrayed themselves as resilient individuals, who actively manage their response to the crisis situation and skillfully navigate between protecting their wellbeing and sustaining climate-friendly behaviours. Building on earlier work (Marczak et al., 2022), we advocate for a change in a perspective on emotions related to climate change by drawing attention to potentially energising and health-preserving emotions that are congruent with values and goals of people concerned about climate change. Furthermore, we describe specific coping strategies that allow our participants to regulate their emotions. Although drawing upon evidence from Poland, we argue that our findings are of universal relevance, as many populations face similar challenges regarding climate change.

As such, the current work can serve as an informed basis for subsequent quantitative studies on emotions related to climate change and their impact on mental health, decision making and climate action taking. For instance, our findings can help to create more reliable psychometric measures to study emotions related to climate change. Furthermore, insights from the current study can inspire future experimental work focused on how distinct emotions translate into pro-environmental behaviour (Brosch, 2021). Apart from that, our work may help policymakers raise awareness of climate change more effectively. Our results clearly indicate that people experience a variety of different emotions, many of which have the potential to engage people with the issue of climate change. Negative emotions likely lead to greater risk perception, greater policy support and have the potential to transform indifference into climate action. Positive emotions, on the other hand, are a source of empowerment, efficacy and have the potential to keep people engaged. While our study underscores the role of emotion in climate communication, more research is needed to understand how to effectively engage different audiences and use emotions in guiding public decisions and behaviour.

## Limitations

While in the current study we conducted interviews with individuals highly concerned about climate change, we did not assess the level of their concern with any standardized, psychometric questionnaire. This can be seen as a potential limitation, since our sampling method relied simply on participants' own judgement of their level of concern. However, a preselection on the basis of an existing tool, for example, Climate Anxiety Scale (Clayton, 2020) would limit the study group to people experiencing a particular set of emotions and exclude participants who experienced other strong emotions related to climate change. Given the exploratory character of the study, we decided not to use any measure of climate-related emotions for the selection of participants. Secondly, the study was conducted in the midst of the COVID-19 pandemic (between March and May 2021), which heavily impacted daily life of people worldwide, Poland included. The healthcare crisis and national lockdowns competed for attention and potentially added to the emotional distress experienced by the interviewees in their everyday life. Some of them saw the pandemic as a consequence of climate change and noted that similar health issues may become more frequent with changing climate. Several participants also talked about the way the pandemic-related lockdowns were beneficial for the climate (e.g. reduced carbon emissions). However, since the COVID-19 pandemic was not the focus of our study, more data would be needed to draw scientifically grounded conclusions.

## Supplementary Information

Full interview guide, as well as excerpts from the interviews (original quotes in Polish, together with their English translations) can be found in the supplementary materials. Below is the link to the electronic supplementary material.

Supplementary file1 (XLSX 71 KB)

Supplementary file2 (DOCX 21 KB)

Supplementary file3 (DOCX 18.9 KB)

## Data Availability

The dataset generated during the current study is not publicly available due to the protection of participant’s anonymity.

## Footnotes

1 Polish Youth Strike for Climate (Młodzieżowy Strajk Klimatyczny, https://www.msk.earth/) is a part of an international movement Fridays for Future: https://fridaysforfuture.org/

2 Extinction Rebellion is a decentralised, international and politically non-partisan movement for Climate and Ecological Emergency: https://rebellion.global/
