# Supplementary material for: A wise person plants a tree a day before the end of the world: coping with the emotional experience of climate change in Poland
Source: Curr Psychol. 2022 Oct 14:1–19. Online ahead of print. doi: 10.1007/s12144-022-03807-3 (PMC9561312; doi:10.1007/s12144-022-03807-3)
Supplement: Supplementary file 2 — Supplementary file2 (DOCX 21 KB) [file 12144_2022_3807_MOESM2_ESM.docx]

**Supplementary Table 1.** Interview guide used in the study. Please note that the order of topics was different in each individual interview, except for topics marked with an asterisk.

| **Main questions** | **Additional questions** |
| --- | --- |
| *** Topic 1:** Overall, personal relationship to climate change. | |
| - What is your motivation to participate in this study? - What is your general attitude towards climate change? | - What do you *think* about climate change? - How do you *feel* about climate change? |
| **Topic 2:** Situations in which emotions experienced towards climate change are particularly strong. | |
| - How do you react to climate change? - In what situations are the emotions you experience towards climate change particularly strong?     *If answering this question is difficult for the interviewee, provide some examples (e.g., media reports, conversations with other people).* | - When was the last time you thought about climate change? - What event that you remember, related to climate change, do you consider the most important? - What situation related to climate change touched you the most? - Can you recall a moment in life when you first learned about or became more interested in climate change? |
| **Topic 3:** Specific emotions experienced towards climate change. | |
| - What specific emotions do you feel in relation to climate change? - Can you name these emotions? | *At this stage, use supplementary materials.* |
| **Topic 4:**  Following vs. avoiding news reports about climate change. | |
| - Where do you find information about climate change? - Do you try to follow news about climate change or do you avoid it? - What specific emotions cause you to follow or avoid news about climate change?     *Depending on the interviewee, focus on questions about following vs. avoiding news reports.* | - How do you keep abreast of climate science? - Do you think that there is enough information available on this topic? - In your view, is it a good thing that information about climate change is widely available? - Do you feel overwhelmed by information about climate change and - if so - how do you cope with it? - How does it affect your emotions and mood? |
| **Topic 5:** The intensity of emotions experienced towards climate change. | |
| - How intense are the emotions you experience in connection with climate change? | - If you compared your emotions *to those experienced by people in general*, would you say the emotions you feel in connection with climate change are more intense or less intense? - If you compared the emotions you experience in connection with climate change *to those you experience in connection with other issues*, would you say the former are more intense or less intense? |
| **Topic 6:** Showing vs. hiding emotions related to climate change. | |
| - How do you show the emotions you feel about climate change? - Is it easy for other people to understand how you feel about climate change or do you keep it to yourself?     *Depending on the interviewee, focus on questions about showing vs. hiding emotions.* | - If you *show* your emotions, how do you do it and in what situations? - If you *hide* your emotions, why do you do it and in what situations? - When was the last time you talked with someone about climate change? |
| **Topic 7:** Emotions related to climate change and their impact on *personal relationships*. | |
| - How does the way you feel about climate change impact your personal relationships? - Who do you talk with about climate change? - Is there anyone you would rather not talk with about climate change? | - How does this topic impact your relationships with family, friends, colleagues, neighbours, other people? - Do other people support you in your concern about climate or do you feel alienated in your views? - What types of social interactions and relationships are sources of support? - What types of social interactions and relationships make you feel alienated? |
| **Topic 8:** Emotions related to climate change and their impact on *everyday life*. | |
| - Do the emotions you feel about climate change impact your daily functioning?     *Depending on the interviewee, ask more specific questions.* | - How do the emotions you feel about climate change impact your daily functioning? - How do the emotions you feel about climate impact your daily duties (e.g., work, school, household chores)? |
| **Topic 9:** Emotions related to climate change and their impact on *physical and/or mental functioning.* | |
| - Do the emotions you feel about climate change impact your health or mood? | *If answering this question is difficult for the interviewee, provide some examples of how climate change may impact:*   - *cognitive functioning (e.g., excessive thoughts about climate change, problems with focusing on other things responsibilities);* - *emotional functioning (e.g., decline in life satisfaction, decline in mood, inability to feel pleasure, general stress, anxiety, guilt);* - *physical functioning (e.g., tiredness, sleeplessness, loss of appetite, increased heart rate, rapid breathing, sweating, restlessness, dizziness).* |
| **Topic 10:** Persistence of symptoms. | |
| *This part is applicable only if the interviewee feels climate change impacts their physical and/or mental health.* | - For how long have you noticed these symptoms? - Do you feel that these symptoms interfere with your everyday functioning? - Do you feel out of control because you experience these symptoms? - Is it usual for you to experience such symptoms (e.g., in other situations) or do you experience them solely in relation to climate change? - Have you sought and/or received professional psychological or psychiatric help due to the symptoms you experience? |
| **Topic 11:** Climate change as a major, life-changing event. | |
| - Do climate change and the way you feel about it affect your life? - Do you feel that your life has changed due to climate change and your concern about this topic? | - What was your life like before you became concerned about climate change? - Can you describe how your attitude towards climate change evolved since you first learned about it? |
| **Topic 12:** Beliefs about the future in the face of climate change. | |
| - What do you expect the future to look like, given climate change? - Do you (or your acquaintances) feel personally threatened by climate change? - What are your greatest concerns related to climate change? - Do you take climate change into account when making plans for the future? - What do you do in order to prepare for the crisis caused by climate change and its consequences? | - How do you cope with your beliefs about the future in the face of climate change? |
| **Topic 13:** Sense of responsibility and agency in the face of climate change. | |
| - Who do you consider responsible for climate change and to what extent (e.g., yourself, other people, institutions, authorities, government)? - How do you see your own responsibility for climate change? - How do you see your agency in the face of climate change? | - How does this impact your emotions? |
| **Topic 14:** Commitment to climate action. | |
| - What individual actions do you take as a result of your concern for the climate (e.g., avoiding travel by plane, turning vegetarian)? - What collective actions do you participate in as a result of your concern for the climate (e.g., climate strikes, educating others about climate change)? | - How does this impact your emotions? - Does this help you cope with the emotions you experience towards climate change? |
| **Topic 15:** Coping with emotions experienced towards climate change. | |
| - How do you cope with the emotions you experience towards climate change?     *If answering this question is difficult for the interviewee, ask about specific situations in which strong emotions were experienced and how the interviewee coped with these emotions in this situation.* | - What else helps you cope with the emotions you experience towards climate change? - What would you recommend to other people who find it hard to cope with their emotions towards climate change? |
| *** Topic 16:** Summary of the interview. | |
| *Make sure that all relevant points were discussed and ask if the interviewee would like to add anything.* | |
| *** Topic 17:** Professional help. | |
| *Provide information on professional help, when it might be advisable to look for it (possible symptoms) and what institutions provide it.* | |
