# Supplementary material for: A wise person plants a tree a day before the end of the world: coping with the emotional experience of climate change in Poland
Source: Curr Psychol. 2022 Oct 14:1–19. Online ahead of print. doi: 10.1007/s12144-022-03807-3 (PMC9561312; doi:10.1007/s12144-022-03807-3)
Supplement: Supplementary file 3 — Supplementary file3 (DOCX 18.9 KB) [file 12144_2022_3807_MOESM3_ESM.docx]

**Supplementary Table 2.** Consolidated criteria for reporting qualitative research (COREQ) checklist.

| **No** | **Item** | **Comment** | **Section reported** |
| --- | --- | --- | --- |
| **Domain 1: Research team and reflexivity** | | | |
| Personal characteristics | | | |
| 1 | Interviewer/facilitator | The interviews were conducted by DZ, MK, AMH, and MW. | Data collection |
| 2 | Credentials | The interviewing team comprised four academics: DZ and MK have masters degree, AMH and MW have doctoral degree. | Positionality and ethics |
| 3 | Occupation | All interviewers were researchers at the time of the study. | Positionality and ethics |
| 4 | Gender | All interviewers are female. | Positionality and ethics |
| 5 | Experience and training | DZ, who coordinated the study, has previous experience in qualitative research. The remaining interviewers had no previous experience in qualitative research. The interviewers completed training and attended weekly supervision sessions. | Data collection |
| Relationship with participants | | | |
| 6 | Relationship established | No relationship was established with participants prior to the interview. | N/A |
| 7 | Participant knowledge of the interviewer | Participants were informed about the objectives of the study prior to the interview and the role and affiliations of the interviewers. | Data collection |
| 8 | Interviewer characteristics | All members of the research team describe themselves as concerned about climate change. | Positionality and ethics |
| **Domain 2: Study design** | | | |
| Theoretical framework | | | |
| 9 | Methodological orientation and theory | It is an exploratory study based upon qualitative thematic analysis. | Data analysis |
| Participant selection | | | |
| 10 | Sampling | Purposive sampling among volunteers who completed the screening survey. | Participants |
| 11 | Method of approach | Participants were approached by e-mail. | N/A |
| 12 | Sample size | 40 participants took part in the study. | Participants |
| 13 | Non-participation | In the case of 10 participants who dropped out from the study (e.g., did not show up for the interview), additional participants were selected for the study. | Participants |
| Setting | | | |
| 14 | Setting of data collection | The interviews were conducted remotely, either via online video conference or by phone. | Data collection |
| 15 | Presence of non-participants | No one else was present besides the participants and researchers. | N/A |
| 16 | Description of sample | Polish-speaking adults who expressed strong concern about climate change were invited to participate in the study. Importantly, we intentionally selected participants with various demographic characteristics to ensure the diversity of perspectives. | Participants |
| Data collection | | | |
| 17 | Interview guide | The complete interview guide is provided by the authors. Each interviewer completed 2-3 pilot interviews prior to data collection. | Data collection |
| 18 | Repeat interviews | No repeated interviews. | N/A |
| 19 | Audio/visual recording | Interviews were audio-recorded. | Data collection |
| 20 | Field notes | Field notes were taken by the interviewers, if necessary. | N/A |
| 21 | Duration | Interviews lasted between 28 to 152 min. | Data collection |
| 22 | Data saturation | Data saturation has been reached. | Participants, Data collection |
| 23 | Transcripts returned | Transcripts were not returned to participants for comment and/or correction. | N/A |
| **Domain 3: Analysis and findings** | | | |
| Data analysis | | | |
| 24 | Number of data coders | All four interviewers contributed to the development of the code system and coded the interview data. Each interview was coded by at least two interviewers. | Data analysis |
| 25 | Description of the coding tree | Authors did not provide a description of the coding tree. | N/A |
| 26 | Derivation of themes | Themes were derived from the data. | Data analysis |
| 27 | Software | MAXQDA 2020 (VERBI Software) was used to manage the data. | Data analysis |
| 28 | Participant checking | Participants did not provide feedback on the findings. | N/A |
| Reporting | | | |
| 29 | Quotations presented | Quotations are presented to illustrate the themes and findings. Quotations are provided in Polish, along with their English translations. Participant gender and age are provided for each quotation. | Results |
| 30 | Data and findings consistent | Data presented and findings are consistent. | Results |
| 31 | Clarity of major themes | Major themes are clearly identified. | Results |
| 32 | Clarity of minor themes | Minor themes are clearly identified | Results |

Developed from: Tong, A., Sainsbury, P., & Craig, J. (2007). Consolidated criteria for reporting qualitative research (COREQ): a 32-item checklist for interviews and focus groups. *International Journal for Quality in Health Care, 19*(6), 349–357. doi: [10.1093/intqhc/mzm042](https://doi.org/10.1093/intqhc/mzm042)
